# A novel contact force sensing pulsed field ablation catheter in a porcine model

**DOI:** 10.1002/clc.24220

**Published:** 2024-01-29

**Authors:** Juan Hua, Qinmei Xiong, Qiling Kong, Liang Xiong, Qianghui Huang, Jinzhu Hu, Juxiang Li, Jianxin Hu, Peng Si, Tuo Zhou, Qi Chen

**Affiliations:** ^1^ Department of Cardiology The Second Affiliated Hospital of Nanchang University Nanchang China; ^2^ Cardiac Electrophysiology R&D Center APT Medical Inc. Shanghai China

**Keywords:** a porcine model, atrial ablation, contact force sensing, pulsed field ablation, ventricular ablation

## Abstract

**Background:**

Pulsed field ablation (PFA) has emerged as a novel non‐thermal modality with highly myocardium‐specific. However, the PFA catheter based on contact force (CF)‐sensing has not been reported. The study aimed to evaluate the efficacy and safety of a novel CF‐sensing PFA catheter.

**Methods:**

First, different CF (5, 15, 25, and 35 g) of the novel PFA catheter were evaluated on lesion dimensions during ablation on right and left ventricle in two pigs. Next, this catheter was further evaluated on four typical sites of superior vena cava (SVC), cavotricuspid isthmus (CTI), right superior pulmonary vein (RSPV), and right inferior pulmonary vein (RIPV) for atrial ablation in another six pigs. Electrical isolation was evaluated immediately after ablation and 30‐day survival. Chronic lesions were assessed via histopathology after euthanasia. Acute and chronic safety outcomes were observed peri‐ and post‐procedurally.

**Results:**

In ventricular ablation, increased CF from 5 to 15 g produced significantly greater lesion depth but nonsignificant increases from 15 to 35 g. In atrial ablation, the novel CF‐sensing PFA deliveries produced an acute attenuation of local electrograms and formation of a continuous line of block in all 6 pigs. The ablation line remained sustained blockage at the 30‐day survival period. The CF of SVC, CTI, RSPV, and RIPV was 9.4 ± 1.5, 14.5 ± 3.2, 17.2 ± 2.6, and 13.4 ± 2.8 g, respectively. Moreover, no evidence of damage to esophagus or phrenic nerve was observed.

**Conclusion:**

The novel CF‐sensing PFA catheter potentiated efficient, safe, and durable ablation, without causing damage to the esophagus or phrenic nerve.

## Graphical abstract

Broadly, there were two parts in this preclinical experiment. In the first stage, we used different doses of contact force (5, 15 , 25, and 35 g) for endocardial ventricular ablation in two pigs to assess the relationship between the CF and lesions. In the second stage, we examined the feasibility and safety of this novel to create an exit block from the superior vena cava or pulmonary vein and assessed its durability after 30 days, along with observation of the potential vulnerability of the esophagus and phrenic nerve. PFA, pulsed field ablation; CF, contact force; SVC, superior vena cava; CTI, cavotricuspid isthmus; RSPV, right superior pulmonary vein; RIPV, right inferior pulmonary vein. RIPV, right inferior pulmonary vein.

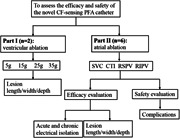

## 1 INTRODUCTION

Atrial fibrillation (AF) is the predominant cardiac arrhythmia encountered in clinical practice, with a high morbidity and mortality and a substantial burden on healthcare.^1^, ^2^, ^3^, ^4^ The ESC, AHA/ACC/HRS and CCS/CHRS guidelines recommend catheter ablation as first‐line therapy in patients with symptomatic AF according to the AF management in 2021.^5^ Currently, there are mainly two types of ablative modalities widely used, including radiofrequency and cryothermal ablation. Although uncommon, there remains severe complications occurred during the procedure, such as pulmonary vein (PV) stenosis, stroke, phrenic nerve palsy, and even atrioesophageal fistula.^6^, ^7^ Among those, most are caused by the thermal energy with propensity to ablate all tissues indiscriminately. In contrast, pulsed field ablation (PFA) has emerged as a novel nonthermal ablative approach for cardiac ablation with highly tissue selectivity, which has also been proven effective and safe since its first clinical application in 2018.^8^, ^9^

As we all know, inadequate contact between the catheter tip and tissue targeted during ablation leads to the generation of edema rather than necrosis, then the subsequent recovery of PV‐left atrium (LA) conduction becomes the chief mechanism of AF recurrence after catheter ablation.^10^, ^11^, ^12^ Contrarily, excessive contact increases the risk of complications such as cardiac perforation, steam pop, and thrombus formation.^13^ Therefore, the appropriate contact force (CF) between catheter tip and the tissue tends to be critical and necessary for successful ablation. With the introduction of CF‐sensing catheters in the early 2010s, these years have witnessed a remarkable and worldwide uptake of CF‐sensing technique, in which the continuous, accurate and real‐time information of CF between the ablation catheter and tissue can be displaying during the procedure.^14^

However, the PFA technique and its related devices are still in a stage of development and lack a uniform standard. As a carrier for releasing energy, the design of the catheter is of great importance, and new devices related to catheter ablation need to be improved and investigated. On this context, we performed this animal experiment to explore the safety and efficacy of a novel PFA system for the first time, including a CF‐sensing catheter and generator, and designed to work with a 3D mapping system. Broadly, there were two parts in this preclinical experiment. In the first stage, we used different doses of CF (5, 15, 25, and 35 g) for endocardial ventricular ablation in two pigs to assess the relationship between the CF and lesions. In the second stage, we examined the feasibility and safety of this novel to create an exit block from the superior vena cava (SVC) or PV and assessed its durability after 30 days, along with observation of the potential vulnerability of the esophagus and phrenic nerve.

## 2 METHODS

### 2.1 Study animals

Eight white pigs (age 4.0 months and 56–62 kg body weight, Shanghai) were included in this study, two of those for ventricular ablation and others for atrial ablation. The study was approved by the Institutional Animal Care and Use Committee (IACUC‐221‐077) and conformed to the Guide for the Care and Use of Laboratory Animals. The animal experiment was performed in the Shanghai Haborside Medical Center Experimental Electrophysiology Laboratory (China). The experimental animals were sedated with propofol (1–8 mg/kg, intravenous), and then the maintenance of general endotracheal anesthesia was through administering inhaled isoflurane (0.5%–5%) after intubation. The entire procedure was strictly sterile and the systemic heparinization was required.

### 2.2 The novel PFA system

The PFA system comprises of three parts: a CF‐sensing catheter (1552002DD06), a proprietary generator (PFA‐micro1, China) and both compatible with the 3D mapping system. Specifically, the novel CF‐sensing catheter has several characteristics (Figure S1): (1) PFA deliveries were set in a single‐point fashion with a speed of 4–10 s/point, much faster than that of RF ablation (30 s/point); (2) It includes four electrodes with a length of 17 mm (the length of each electrode is 2 mm), and D‐2 bipolar discharging is the main output mode; (3) It carries magnetic sensors and matches to HT‐9000Pro 3D navigation system (APTmedical Inc.); (4) It is an optic‐fiber‐based CF‐sensing catheter, and all electrodes have impedance‐based touch index; (5) It has 3‐grade ablative dose for varied depth of lesion: high dose (1800 V/10 trains, lesion depth with 4–7 mm), medium dose (1800 V/5 trains, lesion depth with 3–5 mm), and low dose (800 V/5 trains, lesion depth with 1.5‐3 mm) and; (6) The catheter diameter is 7.5 Fr, compatible with 8.5 Fr adjustable curved sheath for application.

### 2.3 The CF‐sensing PFA catheter for endocardial ventricular ablation

Firstly, the CF‐sensing PFA catheter was placed into the right ventricle (RV). Then, ablation was performed at a discrete position on the RV intima side (to avoid these relative thin areas such as apex and outflow tract). The spatial distance of each position was ≥15 mm, and the number of target locations was more than 10 with high‐dose. The CF was adjusted to 5, 15, 25, and 35 g and the final CF value was checked to be verified maintenance of the targeted values ±3 g. Moreover, the similar operation was performed on the left ventricular (LV) free wall. Next, 2,3,5‐triphenyl tetrazolium chloride (TTC) staining agent was performed intravenously at 3 h after ablation, and euthanasia was performed after 15 min of TTC circulation. Cardiac pacing can be performed through CS catheter during TTC staining agent injection. The width, length and depth of ablation lesion was observed and measured (*n* = 10).

### 2.4 The CF‐sensing PFA catheter for atrial ablation

The procedure was navigated by the 3D system HT‐9000 pro, and the ablation was performed on 4 typical sites of superior vena cava (SVC), cavotricuspid isthmus (CTI), right superior pulmonary vein (RSPV), and right inferior pulmonary vein (RIPV). The ablative dose was chosen based on the thickness of each site. The average thickness of SVC, CTI, RSPV and RIPV was 1–1.5, 4, 2–3, and 3–4 mm, respectively; accordingly, the ablation dose was low, high, medium, and high, respectively (Figure S2). The procedural details were similar as described previously.^15^ Total PFA time was defined as the whole ablation time of PFA delivers.

### 2.5 Efficacy endpoints

The ablation line was assessed using the criteria (voltage amplitude <0.1 mV, and loss capture with pacing the entire length of the line). Specifically, the efficacy of the novel CF‐sensing catheter was evaluated from the following aspects: (1) The acute electrical isolation of SVC or PV was evaluated immediately after ablation. Electroanatomic mapping of SVC, CTI, RSPV and IPV was performed, and whether loss capture during pacing of the entire length of the line was also checked. In addition, ablation parameters and CF value of each site during the procedure were collected and analyzed. (2) The chronic electrical isolation of SVC or PV was re‐evaluated at a 30‐day survival period. (3) Histopathological analysis: gross examination of all hearts was performed at necropsy, and the exterior cardiac surfaces were photographed following euthanasia. Then, all hearts were submitted for histological examination. All specimens were fixed in 10% formalin. The tissue was dehydrated, paraffin embedded, and sectioned at approximately 3–5 μm, and finally stained with Hematoxylin‐Eosin (HE). Lesion continuity, transmurality, depth, and length were measured for each slide.

### 2.6 Safety endpoints

In addition to the efficacy of the CF‐sensing catheter, the safety of this catheter was evaluated from the following aspects: (1) All animals were monitored for their symptoms and signs or other adverse events during the ablation procedure and 30‐day survival period. (2) The phrenic nerve function was evaluated for stimulation threshold in the proximal point of the SVC in six pigs at pre and postablation. Examination of acute phrenic nerve injury was performed by pacing at a strength and duration of 10 mA and 2 ms immediately after the ablation, and it can be gradually increased to 20 mA and 2 ms until the contraction of the diaphragm cannot be induced. (3) SVC, PV and coronary arteries angiography were performed to observe the vessel‐related complications such as stenosis, or dissection at pre‐, post‐ ablation and following 30‐day survival period. (4) The effect of PFA on the esophagus was also evaluated. After inspection of the esophagi, areas with visualized abnormalities were chosen further for histopathological examination.

### 2.7 Statistical analysis

Statistical analyses were performed with SPSS 26.0 software (SPSS Inc.). Continuous variables were presented as the mean with standard deviations, and categorical variables were presented as the frequency and percentage. Statistics were performed using ANOVA and *t*‐tests. *p* < .05 was considered statistically significant.

## 3 RESULTS

### 3.1 Ventricular ablation

**Figure 1:**
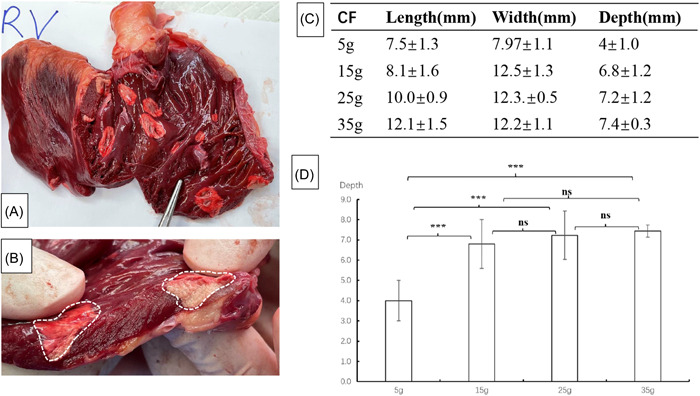
The summary of ventricular ablation. (A) Endocardial aspect of the same lesions in the RV. (B) Inset‐zoomed view of a single lesion. (C) The length, width and depth of different grades of CF. (D) The depth of different grades of CF. CF, contact force; ns, not significant; RV, right ventricle. **p < .01; ***p < .001.

PFA were applied to both the RV (*n* = 10) and the LV (*n* = 10). Lesions were easy to appreciate over the RV endocardium after TTC staining (Figure 1A,B). These lesions demonstrated that the ventricular ablation was successfully performed by this novel PFA catheter. Furthermore, the relationship between different CF and the lesion dimensions was explored. The results indicated that different CF led to different lesion dimensions (Figure 1C). Notably, the lesion depth of varying CF of 5, 15, 25, and 35 g was 4.0 ± 1.0 mm (*n* = 10), 6.8 ± 1.2 mm (*n* = 10), 7.2 ± 1.2 mm (*n* = 10) and 7.4 ± 0.3 mm (*n* = 10), respectively. These results revealed that the CF led to deeper lesion from 5 to 15 g, without significantly deeper lesions from 15 to 35 g (Figure 1D).

### 3.2 Atrial ablation

#### 3.2.1 The acute feasibility and safety of the PFA system

**Figure 2:**
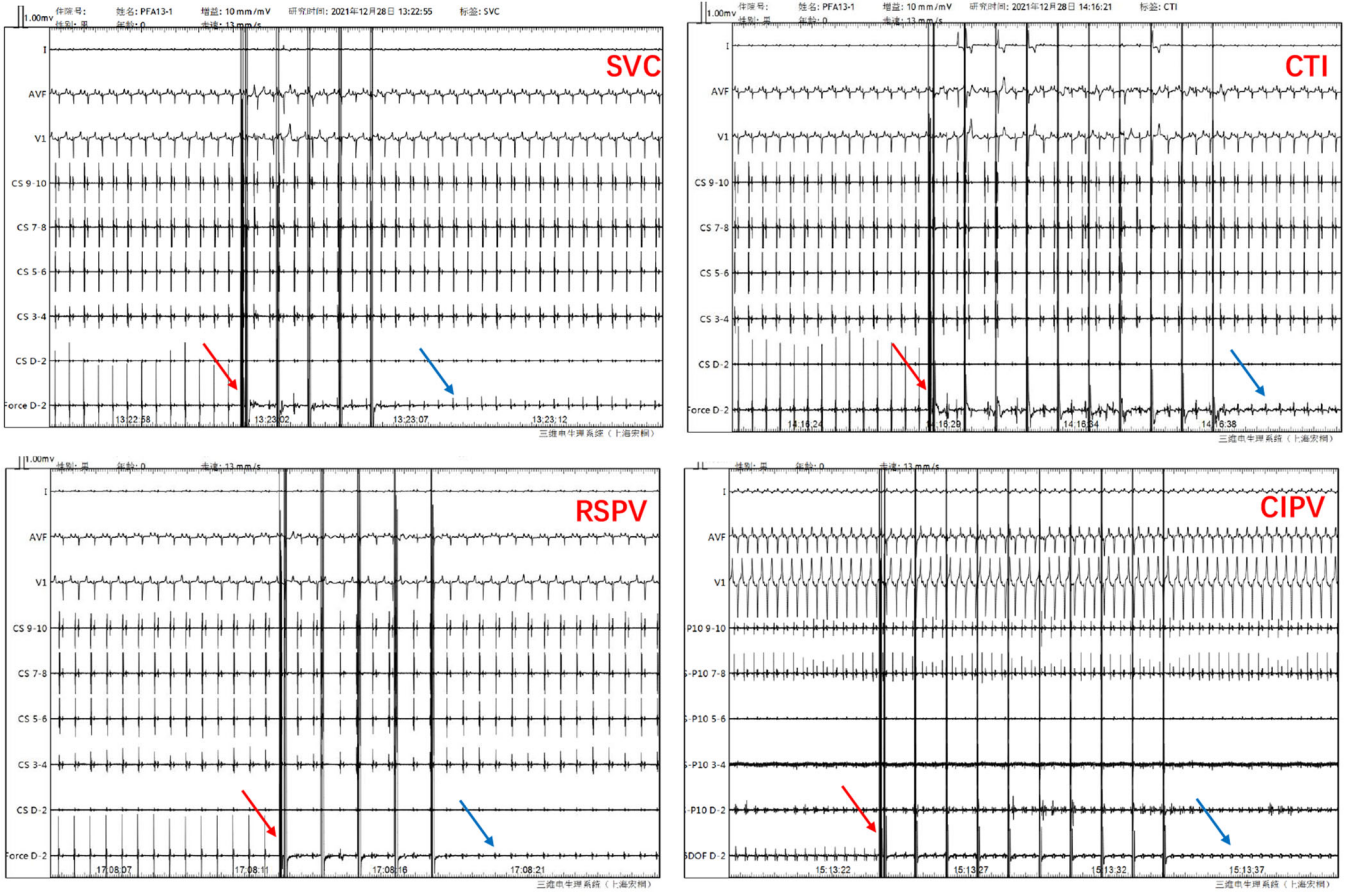
Electrogram attenuation after PFA of each site. Significant electrogram attenuation for each of the targeted sites was observed immediately after PFA. The red arrow indicates the ablation pulse and the blue arrow indicates the decrease of amplitude of tip electrode, respectively. CTI, cavotricuspid isthmus; PFA, pulsed field ablation; RSPV, right superior pulmonary vein; RIPV, right inferior pulmonary vein; SVC, superior vena cava.

**Figure 3:**
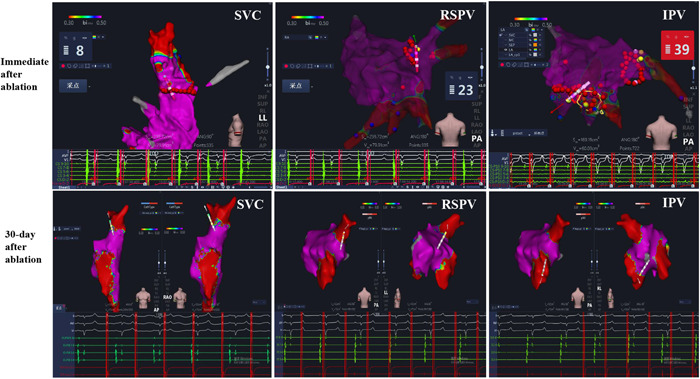
Acute and chronic isolation of the SVC, RSPV, and IPV. This figure shows an example of voltage map immediately after ablation, and following a 30‐day survival period, demonstrating presence of conduction block across the ablation line. IPV, inferior pulmonary vein. The other abbreviations are shown in Figure 2.

The novel PFA system succeeded in establishment of 3D model, mapping of original intracardiac voltage and ablation on parts of SVC, CTI, RSPV, and RIPV in 6 pigs. None of the animals experienced acute serious complications during the PFA deliveries. Totally, 23 PFA applications were successfully delivered to the targeted ablation locations: 6 in the SVC, 6 in the CTI, 6 in the RSPV and 5 in the RIPV. Local electrograms in each of the targeted site were immediately attenuated by after deliveries of PFA (Figure 2), along with loss of capture with pacing in all 6 pigs (Figure 3, upper). The detailed ablation data of ablation in each animal are summarized in Table 1. The ablation time/site of low‐, medium‐ and high‐ dose was 4.5, 5.3, and 9.8 s, and the ablation dose of SVC, CTI, RSPV, and RIPV were low, high, medium, and high, respectively. The ablation applications of SVC, CTI, RSPV, and RIPV were 36.83 ± 10.26, 13.17 ± 6.31, 31.50 ± 11.02 and 49.20 ± 16.59, respectively; and the effective ablation time of was 2.87 ± 0.75, 2.15 ± 1.05, 2.78 ± 0.98, and 8.04 ± 2.54 min, respectively. The CF of SVC, CTI, RSPV, and RIPV was 9.4 ± 1.5, 14.5 ± 3.2, 17.2 ± 2.6, and 13.4 ± 2.8 g, respectively.

**Table 1:** Ablation parameters summary in SCV, CTI, RSPV and IPV.

Ablation location	SVC *N* = 6	CTI *N* = 6	RSPV *N* = 6	IPV *N* = 6
Ablation dose	Low dose	High dose	Medium dose	High dose
Ablation time of each point (sec)	4.5	9.8	5.3	9.8
Ablation applications/site	36.83 ± 10.26	13.17 ± 6.31	31.50 ± 11.02	49.20 ± 16.59
Total PFA time (min)	2.87 ± 0.75	2.15 ± 1.05	2.78 ± 0.98	8.04 ± 2.54
Contact force (g)	9.4 ± 1.5	14.5 ± 3.2	17.2 ± 2.6	13.4 ± 2.8
Activation line of block	6/6	6/6	6/6	5/5
Transmurality	6/6	6/6	6/6	5/5
Lesion depth/mm	1.2 ± 0.3	1.7 ± 0.7	1.4 ± 0.4	2 ± 1.1
Maximum depth/mm	1.6	2.5	1.9	3.8

Abbreviations: CTI, cavotricuspid isthmus; PFA, pulsed field ablation; RIPV, right inferior pulmonary vein; RSPV, right superior pulmonary vein; SVC, superior vena cava.

Furthermore, SVC, PV and coronary angiography were performed immediately after the ablation and no stenosis or dissection was observed in the ablated SVC, PV and nearby coronary arteries (Figures S3 and S4). Additionally, no significant difference on phrenic nerve pacing threshold was observed pre‐ and post‐ ablation (Table S1).

#### 3.2.2 The chronic feasibility and safety of the PFA system

**Figure 4:**
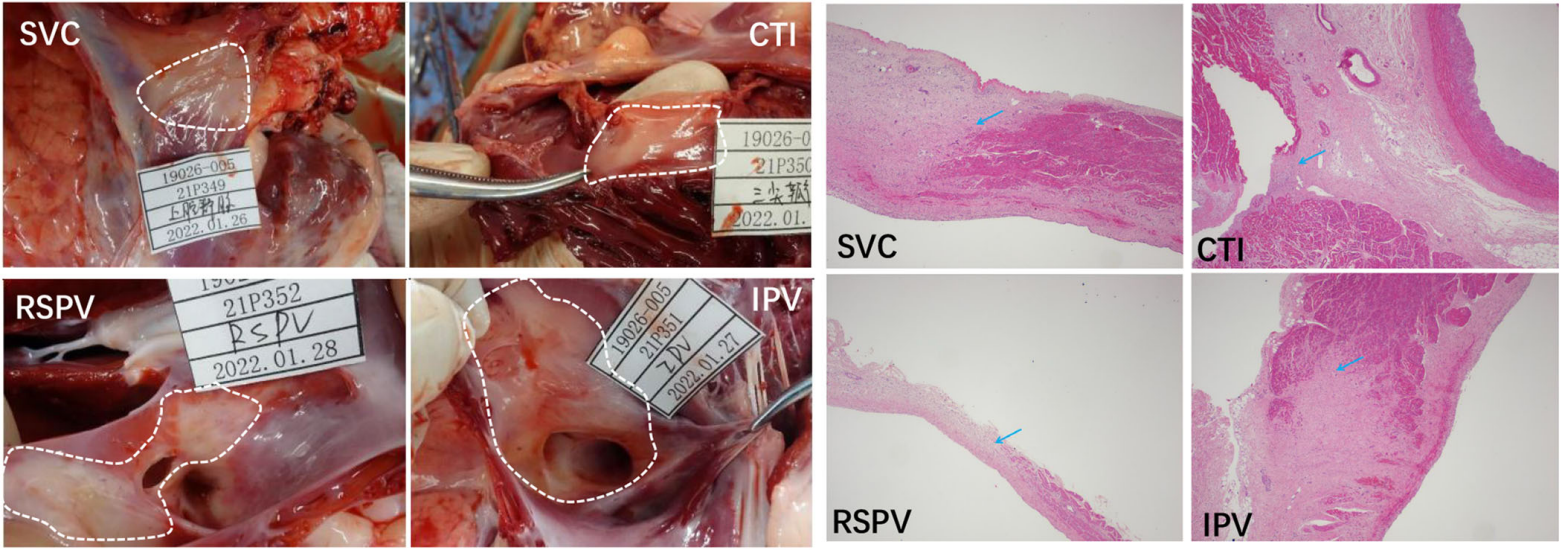
Histopathological examination of PFA in the SVC, CTI, RSPV and IPV. Representative images of a pathological specimen of the SVC, CTI, RSPV and IPV (left). Histopathological examination of above sites conformed transmural replacement fibrosis and showed abrupt transition to normal muscle via HE staining for the indicated sites (right). The blue arrow represented the junction of fibrotic tissues and normal myocardium (H&E, 4×). The abbreviations are shown in Figures 1 and 2.

Furthermore, to verify the lesion duration after the ablation, repeat activation mapping was performed at the 30‐day survival period. Similarly, an example of activation map was shown at a 30‐day survival period and demonstrated the presence of conduction block across the ablation line of SVC and PV (Figure 3, below). In addition, typical lesions were observed in the ablative sites of SVC, CTI, RSPV and IPV (Figure 4, left). Moreover, histopathological results showed a continuous line of transmural fibrosis in 6/6 pigs (Figure 4, right).

During the 30‐day survival period, no animals died and none of the animals presented adverse symptoms of digestive system, such as lack of appetite, dyspepsia, or diarrhea. In addition, no obvious damage to the hematological and plasma biochemical function but a significant Hb drop was observed postablation compared with that of preablation (Table S2). Importantly, no vascular stenosis or dissection in ablated SVC, IPV or SPV was observed through the angiography at 30‐day survival period (Figure S3). Moreover, no evidence of injury was observed in the entire length of the esophageal adventitia and mucosa layers after pathological post‐mortem inspection (Figure S5).

## 4 DISCUSSION

In this preclinical study, we evaluated the efficacy and safety of a novel PFA system. Importantly, the novel catheter was the first to be reported CF‐sensing PFA catheter in ventricular and atrial ablation, which combined the superiority of CF‐sensing catheter and PFA catheter. Firstly, the novel catheter was demonstrated its effectiveness in ventricular ablation, and indicated that CF of 15 g led to greater lesion depth. Next, three different grade‐dose of PFA deliveries were performed to four typical sites of SVC, CTI, RSPV and RIPV in all six pigs, and all resulted in an immediate or chronic changes of exit block (at 30‐day postablation) from the SVC or PV, along with the maintenance of a chronic myocardial and adjacent tissue safety profile.

PFA is a nonthermal ablative modality that preferentially ablates myocardial tissue, which has been used in paroxysmal atrial fibrillation,^8^ persistent atrial fibrillation^16^ and even ventricular arrhythmias in clinical or preclinical trials.^17^ The basis of PFA lies in the application of ultra‐rapid electrical pulses delivered from nanoseconds to microseconds, which generates a strong electrical field that can be targeted towards specific tissue sites.^18^ The strong electrical field delivered induces changes in cellular electrical potential, resulting in the formation of irreversible nanoscale pores and subsequent cellular death.^18^ In addition, the effects of electrical field on cellular properties exhibits tissue specificity, with cardiomyocytes presenting a lower threshold compared to other cells such as blood vessels and nerves. Consequently, PFA merges as a promising candidate for cardiac ablation therapy. No evidence of esophageal injury or phrenic nerve palsy were observed in our study, thus confirming the safety profile of this novel CF‐sensing PFA catheter. However, a significant Hb drop was shown after the ablation, we suspect that may be result from the large number of PFA lesions performed in the animals.

PFA is in an early stage of rapid development, and the development of different instruments is particularly important for the development of this technique. The CF‐sensing catheter reflects the catheter‐tissue contact force in real time by sensing the precise deformation of the micro‐spring between the tip of the connecting catheter and the catheter axis. Therefore, we hypothesized to combine the advantages of PFA catheter and CF‐sensing catheter to explore a better ablation method. Previous studies have shown that the CF information plays a critical role in lesion information during ventricular ablation.^19^, ^20^ In the present study, varying CF 5–15 g resulted in greater lesion depth, without greater lesion depth from 15–35 g. The data may provide some implications about the appropriate CF of the novel catheter in ventricular ablation. This result may be related to the ablative energy, contact time, and catheter orientation, but the exact mechanism for the improvement in lesion depth needs further investigation. In addition, the novel catheter has shown its feasibility and efficacy of AF ablation in four typical sites for atrial ablation. Furthermore, these data indicated that the PFA ablation time of SVC, CTI and RSPV was shorter than that of RIPV, which indicated that the ablation was higher‐efficient in SVC, CTI and RSPV than that of RIPV. A recently published study on contact force PFA system such as “CENTAURI” system^21^ has shown that the Standard wide antral circumferential ablation (WACA) of “CENTAURI” system was performed with a target CF of ≥5 g, and the PVI time was 61.8 ± 23.03 min.^21^ Compared with the “CENTAURI” system mentioned in the above study,^21^ our PFA system has a wider range of CF value and shorter ablation time. Additionally, we have summarized the lesions data of each site, which could provide some references for safely using the catheter in subsequent clinical application. However, due to the different research objects, the differences between these two PFA systems remain to be further confirmed.

PFA can currently be delivered through various catheter designs. Certain catheters are designed to deliver PFA in a single‐shot manner (Fara‐pulse Inc.), while others are designed to change the shape according to the need of application, such as the Farawave ablation catheter (Farapulse, Inc.), the Sphere‐9 ablation catheter (Afera Inc.) and the Pulse Select™ (Medtronic).^22^ Similarly, another multielectrode catheter was evaluated and confirmed it ablation capabilities.^23^ It is well known that a circular mapping catheter is used routinely for LA mapping during PVI and will be time and cost effective, that the routine implementation of a circular mapping catheter for LA mapping during PVI proves to be a time and cost‐effective method. However, the PV anatomy variability is remarkable and, because of this, the use of a basket or flower catheter may present some limitations. In this context, the advantages of linear catheter delivery of PFA in a single‐shot fashion will become more pronounced. In our study, PV isolation was achieved in nearly all animals, regardless of the PV anatomy. The superiority of CF‐sensing catheters can be ascribed to two factors: first, the CF‐sensing catheter in our study is a linear one which delivers of PFA in a sing‐shot manner with high‐accuracy and; second, the catheter tip orientation could be adjusted according to the targeted CF, which may be more efficient and safer compared with other shapes of catheter. Furthermore, the ablation targeted sites of SVC, CTI, RSPV and IPV were more than a prior preclinical study of SVC, RAA, and RSPV,^15^ which will provide more data for the clinical use of the novel PFA system.

This study has several potential limitations. First, the study was performed in a small sample size. Second, this is a preclinical experiment in porcine model, differences are existed in structure between human and porcine anatomy and their susceptibility to field exposures. Furthermore, we did not provide the correlation between the CF and the width, depth of the lesions, which will be the key point of our subsequent work. Further investigations are required regarding to the optimal parameters for PFA, application scope and long‐term safety, which will help this modality more applicable.

## 5 CONCLUSION

In this preclinical model, the novel CF‐sensing PFA catheter potentiated efficient, safe, and durable ablation in four typical sites of SVC, CTI, RSPV, and IPV, and produced durable lesions while minimizing the vulnerability to esophagus or phrenic nerve damage.

## CONFLICT OF INTEREST STATEMENT

The authors declare that they have no conflict of interest.

## Supporting information

Supporting information.

## Data Availability

The data that support the findings of this study are available from the corresponding author upon reasonable request.
