# Supplementary material for: A novel contact force sensing pulsed field ablation catheter in a porcine model
Source: Clin Cardiol. 2024 Jan 29;47(2):e24220. doi: 10.1002/clc.24220 (PMC10823559; doi:10.1002/clc.24220)
Supplement: Supplementary file 1 — Supporting information. [file CLC-47-e24220-s001.docx]

**Supplementary materials**

**A novel contact force sensing pulsed flied ablation catheter in a porcine model**

**Table S1** Phrenic nerve pacing threshold summary

| Animal | Pre-ablation/mA | Post- ablation/mA | *P* value |
| --- | --- | --- | --- |
| 1 | 3 | 4 | 0.61 |
| 2 | 2 | 1 |  |
| 3 | 0.5 | 0.5 |  |
| 4 | 4 | 3 |  |
| 5 | 4 | 4 |  |
| 6 | 0.5 | 0.5 |  |

**Table S2** The hematological and plasma biochemical changes of pre- and post-ablation

| Parameters | Pre-ablation  (N=6) | Post-ablation  (N=6) | *P* value |
| --- | --- | --- | --- |
| RBC | 7.10±1.03 | 5.27±1.09 | 0.014 |
| HGB | 12.53±1.18 | 9.08±2.09 | 0.006 |
| PLT | 389.00±97.11 | 387.17±119.52 | 0.997 |
| WBC | 21.30±3.44 | 17.09±1.47 | 0.020 |
| NEUT | 9.65±2.74 | 5.99±2.35 | 0.032 |
| LYMPH | 9.66±1.50 | 9.42±2.73 | 0.854 |
| ALT | 43.33±7.28 | 57.17±17.05 | 0.098 |
| BUN | 4.17±1.17 | 10.00±3.35 | 0.002 |
| CR | 2.10±0.27 | 2.28±0.40 | 0.375 |

Abbreviations: RBC, red-blood-cell; HGB, hemoglobin; PLT, platelet; WBC, white-blood-cell; NEUT, neutrophil; LYMPH, lymphocyte; ALT, alanine transaminase; BUN, urea nitrogen; CR, creatinine.

**Figures**


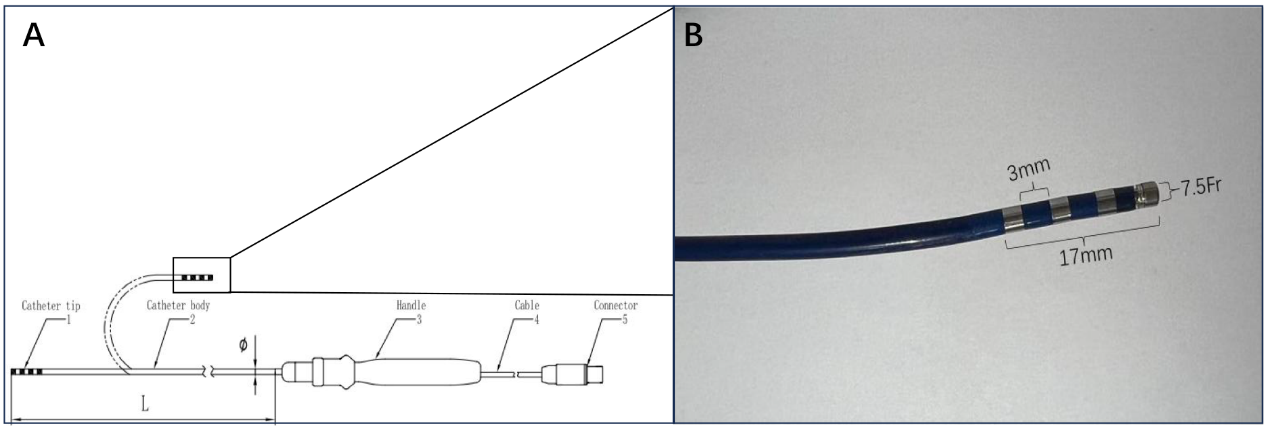


**Figure S1.** Description of the catheter. A: The overall schematic diagram of the catheter. B: Distal aspect of the ablation catheter. The CF-sensing PFA catheter is a linear multielectrode catheter with a dimeter of 7.5Fr. It includes 4 electrodes with a total length of 17mm. The length of each electrode is 2mm. PFA, pulsed field ablation; CF, contact force.


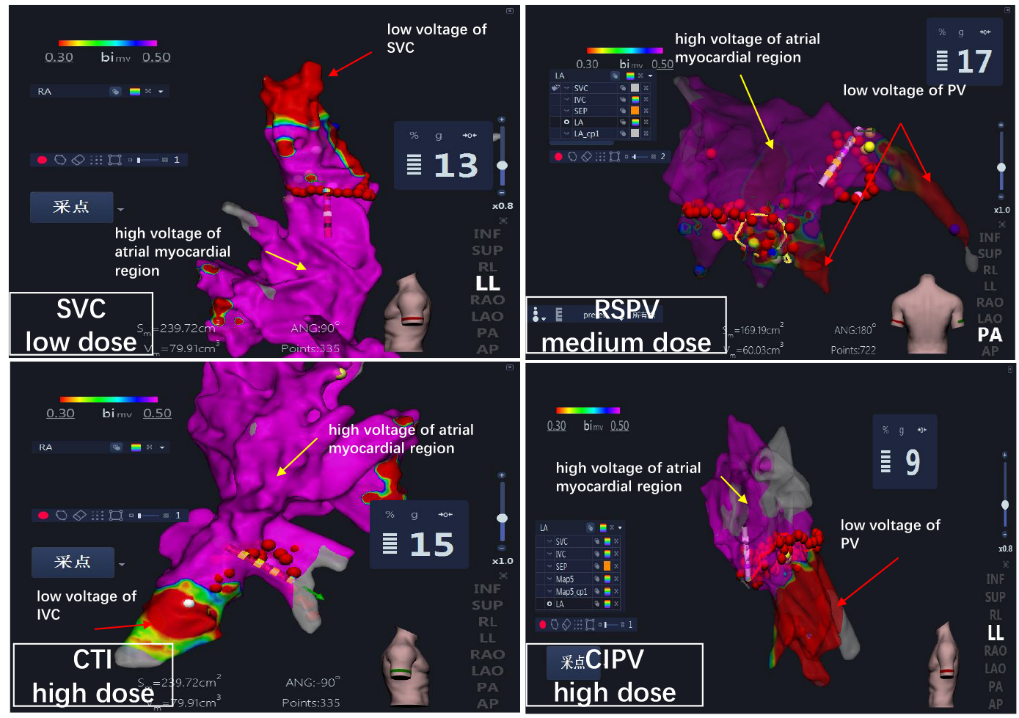


**Figure S2.** Porcine model summary of atrial ablation. In six pigs, a low dose of PFA was delivered to the SVC, a medium dose was delivered to the RSPV, and a high dose was delivered to CTI and common IPV, respectively. SVC, superior vena cava; CTI, cavotricuspid isthmus; RSPV, right superior pulmonary vein; RIPV, right inferior pulmonary vein. Other abbreviations are shown in Figure 1.


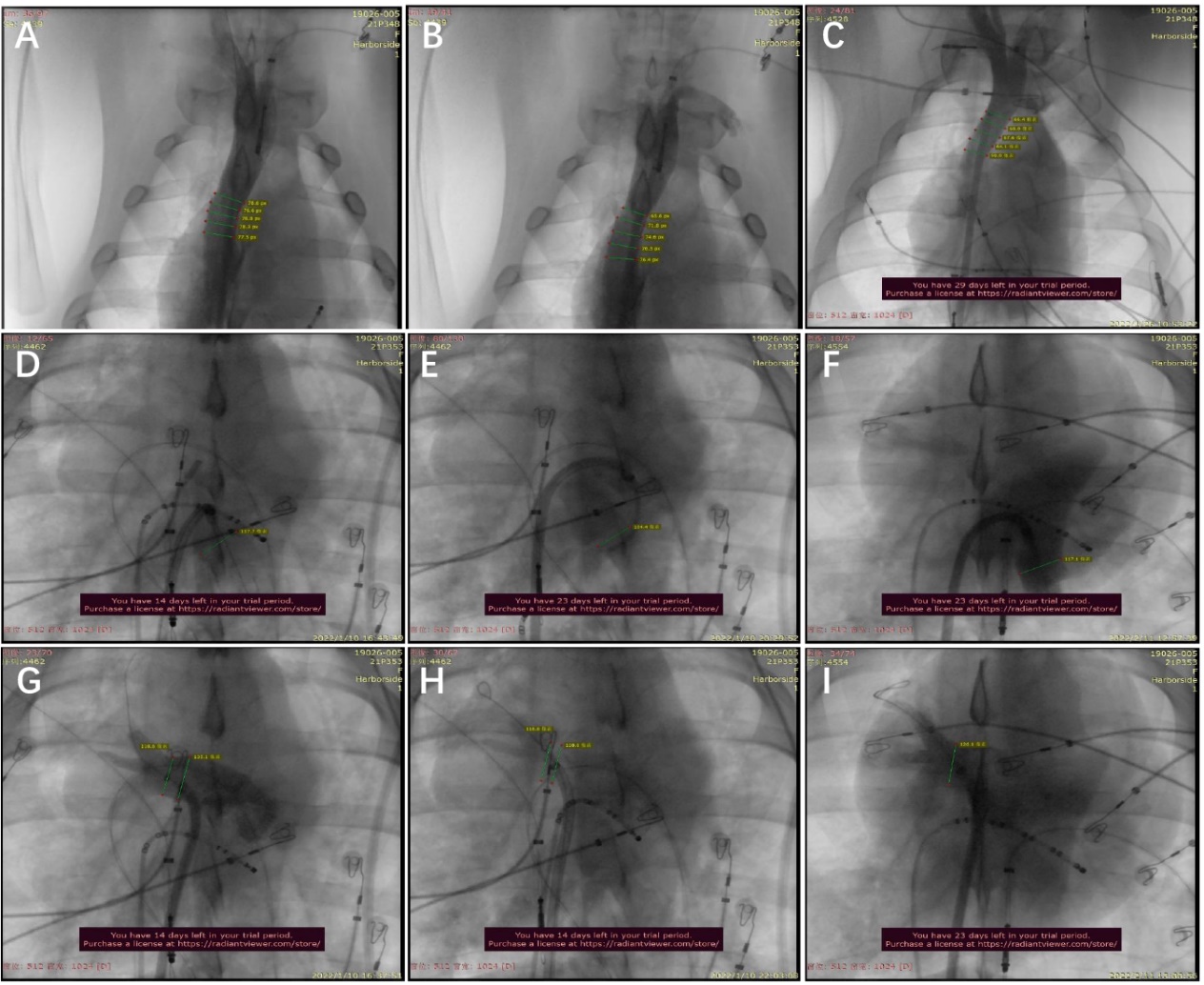


**Figure S3.** SVC, IPV and SPV angiography immediately after ablation. SVC angiography: pre- (A), post-ablation (B) and 30-day survival period (C); IPV angiography: pre- (D), post-ablation (E) and 30-day survival period (F); SPV angiography: pre- (G), post-ablation (H) and 30-day survival period (I). SVC, superior vena cava; IPV, inferior pulmonary vein; SPV, superior pulmonary vein.


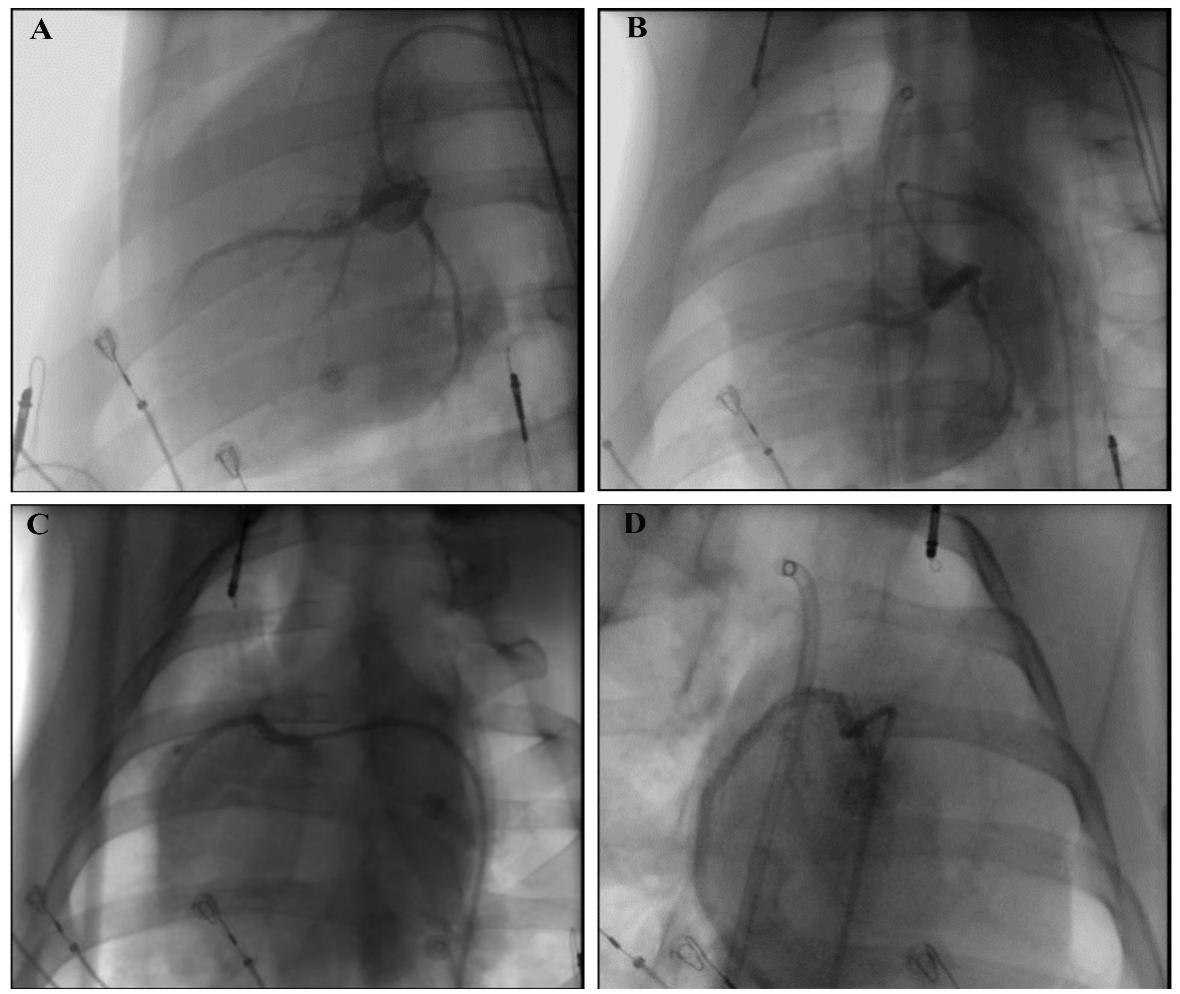


**Figure S4.** Coronary artery angiography immediately after ablation. Left coronary artery angiography: pre- (A) and post-ablation (B); Right coronary artery angiography: pre- (C) and post-ablation (D).


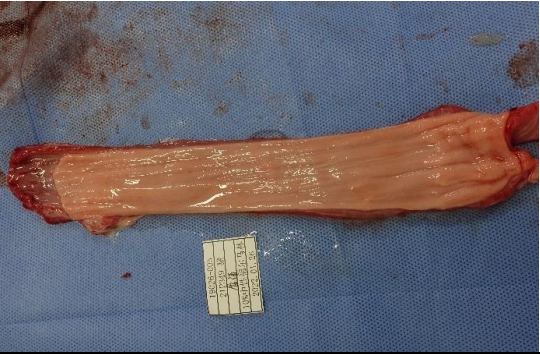


Figure S5. Gross examination of esophageal. The result presented that no damage to the esophagus was shown after ablation.
